# Neuropathologic analysis of Tyr69His TTR variant meningovascular amyloidosis with dementia

**DOI:** 10.1186/s40478-015-0216-0

**Published:** 2015-07-10

**Authors:** Jennifer L. Ziskin, Michael D. Greicius, Wan Zhu, Anna N. Okumu, Christopher M. Adams, Edward D. Plowey

**Affiliations:** Department of Pathology, Stanford University School of Medicine, Edwards Building, Room R-241, 300 Pasteur Drive, Stanford, CA 94305 USA; Department of Neurology and Neurological Sciences, Stanford University School of Medicine, Stanford, CA 94305 USA; Vincent Coates Foundation, Stanford University Mass Spectrometry, Stanford, CA 94305 USA

**Keywords:** Transthyretin, Amyloid, Tau/MAPT, Dementia

## Abstract

*Transthyretin/TTR* gene mutations usually cause systemic amyloidotic diseases. Few *TTR* variants preferentially affect the central nervous system, manifesting as oculoleptomeningeal amyloidosis. Patients with TTR meningovascular amyloidosis often show dementia, however the neuropathologic features of dementia in these cases have not been elucidated. We report the neuropathologic findings from a brain autopsy of a 72-year-old man with the rare Tyr69His (Y69H) *TTR* gene variant, dementia and ataxia. Severe amyloid deposits were observed in the leptomeninges and in a subpial and subependymal distribution. Mass spectrometry analysis demonstrated that the amyloid deposits were comprised of over 80 % of the variant TTR. TTR was undetectable by mass spectrometry in the neocortex subjacent to the subpial amyloid deposits. Subpial TTR amyloid deposits were associated with brisk superficial reactive gliosis and siderosis in the neocortex and cerebellar cortex. Subependymal TTR amyloid deposits were associated with subjacent myelin pallor in the hippocampal outflow tract structures including the alveus, fimbria and fornix. Phospho-tau immunostains demonstrated transentorhinal-stage neurofibrillary degeneration (Braak stage II) which, in the absence of neocortical amyloid-beta and neuritic plaques, was indicative of primary age-related tauopathy (PART). However, distinctive phospho-tau aggregates were observed subjacent to the subpial TTR amyloid deposits in all regions of the neocortex, including the primary motor and striate cortices, suggesting a potential link between TTR amyloid and neocortical tauopathy. Our report reveals novel insights into the potential neuropathologic substrates of dementia in variant TTR amyloidosis that need to be investigated in larger autopsy series.

**Electronic supplementary material:**

The online version of this article (doi:10.1186/s40478-015-0216-0) contains supplementary material, which is available to authorized users.

## Background

Transthyretin (TTR) is a soluble protein tetramer that carries thyroxine and retinol binding protein in the circulation [1]. Insoluble TTR amyloid deposition is most commonly seen as a systemic disease in elderly individuals [2]. Patients with *TTR* mutations also most often present with systemic amyloidotic diseases including familial amyloidotic polyneuropathy (FAP) [3] and familial amyloid cardiomyopathy [2]. Mild cerebral TTR amyloid angiopathy and choroid plexus amyloidosis can be observed in systemic amyloidotic diseases like FAP [4, 5]. In contrast, severe meningovascular amyloidosis is associated with certain *TTR* gene mutations, including Leu12Pro [6], Asp18Gly [7, 8], Ala25Thr [9, 10], Val30Gly [11–13], Val30Met [14, 15], Thr49Pro [16], Leu58Arg [17], Phe64Ser [18], Tyr69His [19–21] and Tyr114Cys [22], and can lead to dementia and ataxia. To gain potential insights into the pathogenesis of these deficits in meningovascular amyloidosis, we report our postmortem neuropathologic findings from a patient with dementia, ataxia and the rare Tyr69His (Y69H) *TTR* substitution.

## Patient and methods

Detailed methods can be found in Additional file 1: Supplemental Methods.

### Ethics, consent and permissions

Informed consent to publish the results of this autopsy study was obtained from the patient’s next of kin.

## Case presentation

A 72-year-old Italian-American male demonstrated progressive cognitive decline over 13 years punctuated by multiple encephalopathic episodes that included headache, confusion, ataxia and short-term memory loss. Six years prior to death, radiographic workup revealed superficial siderosis and an arteriovenous malformation involving the thoracolumbar spinal cord (T11), findings which were previously reported [23]. Resection of the arteriovenous malformation alleviated the patient's encephalopathic episodes but did not ameliorate his moderate ataxia nor halt his progressive cognitive decline. A follow-up visit three months after his resection was notable for severe cognitive impairment. He scored a 9 on the 30-point mini-mental state examination with deficits in language, memory, executive function, and visuospatial skills. A complete autopsy demonstrated that the patient died of aspiration pneumonia, sepsis and multiple organ system failure. Mild to moderate amyloidosis was also noted in the systemic organs examined histologically (Additional file 1: Table S1). A standard dementia neuropathologic workup [24] was performed.

### Histopathology

Tissue sections (6 μm thickness) were stained with hematoxylin and eosin. Immunoperoxidase reactions with the following antibodies were performed with standard methods: α-synuclein (Cell Signaling #2642, 1:1000); amyloid-beta (clone 6F/3D, Dako, M0872, 1:400; clone 4G8, BioLegend, SIG-39220, 1:500); Fused in sarcoma (FUS; Sigma, HPA008784, 1:3000); glial fibrillary acidic protein (GFAP; Dako, Z0334, 1:2000); myelin basic protein (Dako A0623, 1:400); phospho-MAPT (clone AT8; Thermo Scientific, MN1020, 1:2000); 3-repeat isoform MAPT (3R MAPT, RD3; clone 8E6/C11, Millipore, 05–803, 1:250); 4-repeat isoform MAPT (4R MAPT, RD4; clone 1E1/A6, Millipore, 05–804, 1:250); TDP-43 (Proteintech 10782-2-AP, 1:10,000); TTR (Dako A0002, 1:4000). Special stains, including the modified Bielschowsky stains, Gallyas silver stains, Luxol fast blue-periodic acid Schiff stains, were also performed. Formalin-fixed, paraffin-embedded (FFPE) tissues were sampled and reprocessed for transmission electron microscopy (TEM) using standard techniques.

### Molecular analyses

*APOE* genotyping was performed on genomic DNA extracted from FFPE tissue sections via restriction fragment analysis according to the method of Kamboh and colleagues [25]. All 4 exons of the patient’s *TTR* gene were sequenced from the same genomic DNA. Tissue cores (3 mm diameter; 0.8 to 1.7 mg) punched from the formalin-fixed, paraffin embedded tissue blocks were analyzed by mass spectrometry (MS) [26].

## Results

**Fig. 1:**
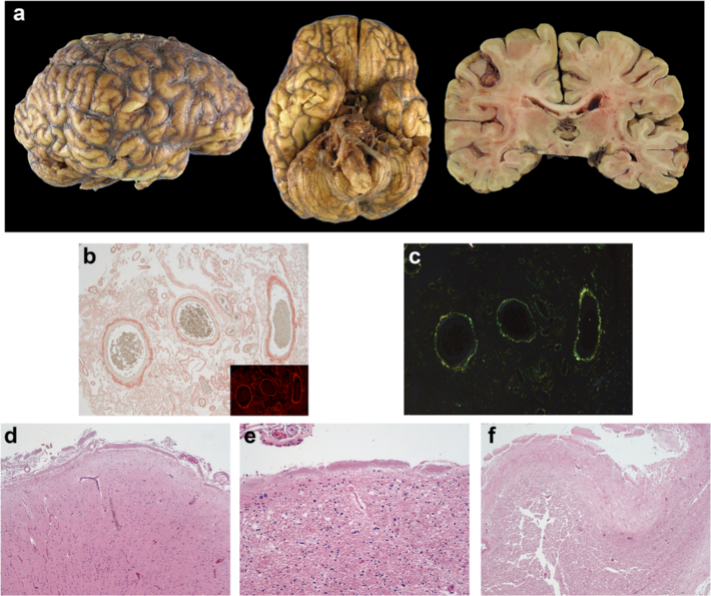
Meningovascular and ventricular amyloidosis associated with Tyr69His substitution in TTR. **a** Gross images demonstrating golden-brown discoloration of the leptomeninges of the cerebral hemispheres and the cisterns at the base of the brain. A coronal section of the brain demonstrates diffuse cortical atrophy, but very little hippocampal atrophy or ventricular dilatation. **b** A Congo red stain performed on the cerebral leptomeninges highlights the vascular and extravascular amyloid deposits (inset, Congo red under epifluorescence microscopy with Texas red filter; original magnifications of 40×). **c** Visualization of the Congophilic meningovascular amyloid under polarized light reveals the diagnostic apple-green birefringence (Congo red; original magnification of 40×). **d** Subpial amyloid deposits in the insular cortex (H&E; original magnification of 40×). **e**, **f** Subependymal amyloid deposits in the alveus (**e**; H&E; original magnification of 100×) and fornix (**f**; H&E; original magnification of 40×)

Gross examination of the 1390 g brain revealed golden-brown discoloration and hardening of the leptomeninges over the cerebral hemispheres and cisterns at the base of the brain (Fig. 1a). There was mild cortical atrophy involving the frontal, temporal and parietal lobes, the insula and the cerebellar vermis. There was minimal atrophy of the hippocampus and no significant hydrocephalus *ex vacuo*. Histologic sections of the leptomeninges revealed severe congophilic vascular and extravascular amyloid deposits (Fig. 1b,c) with minimal extension along cortical penetrating vessels. Granulomatous angiitis was not seen. In the basilar cisterns, the vascular amyloid was associated with moderate to severe vasculopathy with smooth muscle loss and double barrel vascular profiles. Extensive subpial amyloid was identified in the cerebral hemispheres (Fig. 1d) and superficial cerebellar folia. Extensive intraventricular congophilic amyloid aggregates and subependymal amyloid deposits involving the alveus (Fig. 1e) and fornix (Fig. 1f) were seen.

We employed the immunostains at our disposal, Amyloid-beta and TTR, to attempt to identify the amyloid protein in FFPE tissue block sections with negative results (Additional file 1: Figure S1a, b). However, TEM images demonstrated leptomeningeal amyloid fibrils that resemble previously reported TTR amyloid fibrils (Additional file 1: Figure S1c) [27]. We subsequently employed MS-based proteomic analysis on core samples of the amyloid-laden leptomeninges harvested from the paraffin blocks. The most abundant protein identified by MS in the meningovascular amyloid samples was TTR (Additional file 1: Figure S2a). Extracted ion chromatograms (EICs) demonstrated that mutant Y69H TTR comprised over 80 % of the of TTR peptides T49-K70. TTR was not detected in the control brain parenchyma samples from the basis pontis and subcortical white matter. Furthermore, TTR was not detected in a sample of the insular cortex, demonstrating that amyloidogenic TTR does not penetrate past the subpial space into the cortical parenchyma. We sequenced the patient’s *TTR* gene exons and detected a heterozygous T to C point mutation in the first nucleotide position of codon 69, exon 3, which encodes for the amino acid substitution of tyrosine for histidine (Additional file 1: Figure S2b). Exons 1, 2 and 4 demonstrated wild type sequences. The patient’s *APOE* genotype was determined to be E2/E3 (Additional file 1: Figure S2c).

**Fig. 2:**
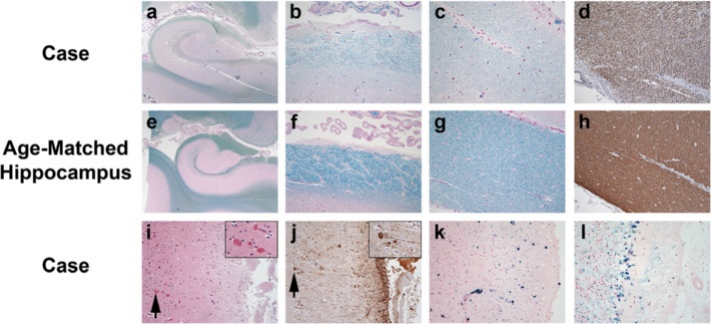
Evidence of subependymal and subpial injury. Sections of hippocampal efferent tracts from our patient (**a**-**d**) were compared to age-matched control cases (**e**-**h**). Luxol fast blue (LFB) stains demonstrate myelin pallor in Ammon’s horn (**a**, **e**; LFB; original magnification of 10×), the alveus (**b**, **f**; LFB; original magnification of 100×) and fimbria (**c**, **g**; LFB; original magnification of 100×) in TTR amyloidosis compared to the age matched hippocampus. Myelin basic protein (MBP) immunostains demonstrated similar myelin pallor in the fimbria (**d**, **h**; MBP; original magnification of 100×). **i**. The superficial insular cortex demonstrated gliosis with eosinophilic bodies (arrows; H&E; original magnification of 100×), similar to those reported in Herrick et al. [14], that were variably GFAP immunoreactive (**j**; GFAP; original magnification of 100×). Iron stains demonstrated siderosis in the upper cortical layers of the superior temporal gyrus (**k**; iron stain; original magnification of 200×) and in the Bergmann glia of the atrophied cerebellar vermis (**l**; iron stain; original magnification of 200×)

We further investigated the brain for neuropathology to explain his dementia. We hypothesized that the prominent subependymal amyloidosis (Fig. 1e,f) might damage hippocampal efferent tracts that line the ventricles, including the alveus, fimbria and fornix. Special stains for myelin, including LFB/PAS and myelin basic protein immunohistochemistry, demonstrated severe myelin pallor in the alveus and fimbria compared to 2 age-matched autopsy brains with no significant neuropathology (Fig. 2a-h). There was no histologic evidence of subcortical leukoencephalopathy and only a single, minute, remote cortical infarct was seen in a section of the right postcentral gyrus (Additional file 1: Figure S5f). The lack of ischemic pathology is consistent with pre-mortem 3 T magnetic resonance FLAIR images showing no obvious white matter changes or microinfarcts (Additional file 1: Figure S3a). We observed brisk gliosis in the upper neocortical layers subjacent to the subpial amyloid deposits (Fig. 2i,j). These findings suggest that subependymal and subpial TTR amyloid deposits are associated with injury to the subjacent brain parenchyma, including hippocampal efferent tracts and superficial layers of the neocortex. Iron deposits were observed histologically in the superficial neocortex, especially in sections of the frontal and temporal lobes (Fig. 2k), and in the Bergmann glia of the atrophied Purkinje cell layer of the vermis (Fig. 2l). Pre-mortem GRE images also demonstrate the superficial siderosis (Additional file 1: Figure S3b). Recent or remote microvascular hemorrhages were not seen.

**Fig. 3:**
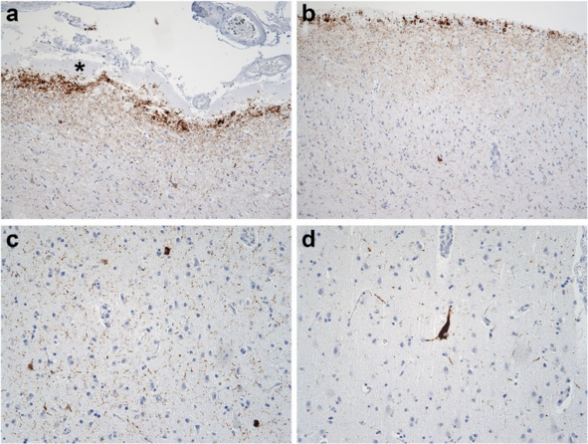
Cortical tauopathy in TTR meningovascular amyloidosis. Phospho-MAPT (AT8) immunostains were performed to determine if TTR meningovascular amyloidosis and dementia were associated with tauopathy. **a**, **b** Intense granular, threadlike and globular AT8 immunoreactivity was observed in the molecular layer of the entorhinal cortex (**a**; AT8; original magnification of 100×) and frontal neocortex (**b**; AT8; original magnification of 100×) subjacent to subpial amyloid deposits (asterisk). **c** AT8ir threads and neurons were observed in deep layers of the frontal neocortex (AT8; original magnification of 200×). **d** Rare neocortical neurofibrillary tangles were also seen (AT8; original magnification of 200×)

Phospho-MAPT immunostains (clone AT8) revealed neurofibrillary tangles in Pre α cells of the entorhinal cortex and focally moderate neurofibrillary tangles in the pyramidal cells of the hippocampal CA1 sector, indicative of Braak stage II or B1 transentorhinal stage of neurofibrillary degeneration (Additional file 1: Figure S4). Although there were conspicuous AT8 immunoreactive (AT8ir) threads surrounding the vestigial hippocampal sulcus (Additional file 1: Figure S4c), we saw no tangles in CA2 or the fascia dentata. Curiously, we also observed neocortical tauopathy characterized by AT8ir threads and neuronal somata. Most distinctive were subpial AT8ir granules, globules and threads subjacent to the subpial TTR amyloid deposits throughout the neocortex (Fig. 3a,b). AT8ir threads and neurons and rare neurofibrillary tangles were also found in the deeper levels of the neocortex (Fig. 3c,d). Subpial and neocortical tauopathy was most prominent in a section of the middle frontal gyrus (Fig. 3), but was present throughout the neocortex including primary motor and striate cortices (Additional file 1: Table S2). The parenchymal threads and most of the AT8ir neurons were not argyrophilic (Gallyas stains, not shown). Scattered axons in the subcortical white matter also demonstrated AT8 immunoreactivity, but there was no apparent tauopathy in glial somata.

Immunostains for amyloid-beta (clones 6F3D and 4G8) demonstrated a complete absence of amyloid-beta plaques in the neocortex (Additional file 1: Figure S5a). Bielschowsky stains likewise revealed an absence of neocortical neuritic plaques. We incidentally observed focal sparse neuritic plaques, immunoreactive for amyloid-beta and AT8, in the stratum oriens of the hippocampal CA1 sector (not shown). These few focal neuritic plaques were distinct from the TTR amyloid deposits in the alveus. Grade I cerebral amyloid-beta angiopathy was seen in only a few leptomeningeal vessels with the 4G8 antibody (Additional file 1: Figure S5b) [28]. Immunostains for α-synuclein (Additional file 1: Figure S5c), TDP-43 (Additional file 1: Figure S5d) and FUS (Additional file 1: Figure S5e) revealed no abnormal neuronal inclusions.

## Discussion

Several TTR substitutions are associated with biopsy- or autopsy-proven meningovascular amyloidosis [6–22]. Many patients with these mutations suffer from dementia and/or ataxia. Previously, Blevins and colleagues [19] reported only sparse neocortical neuritic plaques and amyloid-beta plaques and no evidence of hemorrhages or siderosis in a non-demented patient from a Swedish kindred with the Tyr69His TTR substitution. Post-mortem neuropathologic dementia workups in cases of meningovascular TTR amyloidosis with dementia have not been reported in the literature. The neuropathologic underpinnings of meningovascular amyloidosis-associated dementia are therefore unknown.

To provide novel insights into the potential neuropathologic substrates of dementia and ataxia in this disorder, we present the postmortem neuropathologic findings from a patient with dementia, ataxia and meningovascular amyloidosis associated with the rare Tyr69His (Y69H) substitution in TTR. To our knowledge, our patient, whose maternal and paternal ancestors originated from northwest Italy, is not related to the prior 2 reported kindreds with the Tyr69His TTR substitution from Sweden and Saskatchewan [19–21]. Our histologic and MS data demonstrate that TTR amyloid does not penetrate the cortex and subcortical white matter. Rather, TTR amyloid is deposited in leptomeningeal vessels and in subpial and subependymal deposits. This observation strongly argues that injuries to periventricular structures and superficial cortex are likely relevant for the neurologic deficits. We observed subependymal TTR amyloid and myelin loss in hippocampal efferent tracts including the alveus, fimbria and fornix that may underlie our patient’s memory deficit. We also observed brisk reactive gliosis and dystrophic astrocytic processes, which were previously noted by Herrick and colleagues [14], in the superficial neocortex. Our findings may implicate neuronal toxicity subjacent to subpial and subependymal TTR amyloid deposits in the pathogenesis of the dementia and ataxia.

A novel finding in this report is the association of neocortical tauopathy with meningovascular TTR amyloidosis. AT8 immunostains revealed Braak stage II [29], or transentorhinal B1 stage [24], of neurofibrillary degeneration based on the presence of neurofibrillary tangles in the Pre α neurons of the entorhinal cortex and only a few neurofibrillary tangles in the CA1 sector of the hippocampus. These neurofibrillary tangles were immunoreactive for both 3R and 4R MAPT and appeared to have paired-helical filament ultrastructure. In our patient, whose *APOE* genotype was E2/E3, there were no neocortical neuritic plaques, no neocortical Aβ deposits and no hippocampal granulovacuolar degeneration. We identified only focal sparse neuritic plaques of uncertain significance in the stratum oriens of the hippocampal CA1 sector. In the absence of neocortical amyloid-beta and neuritic plaques in our patient, the transentorhinal stage neurofibrillary degeneration is indicative of early primary age-related tauopathy (PART) [30].

Interestingly, we found AT8ir threads and neurons in nearly all of the neocortical regions examined, including the primary motor cortex and striate cortex, which are classically considered the last to be affected by tauopathy in PART and Alzheimer disease (AD). The density of threads and neurons appeared increased over the levels typically seen with Braak stage II PART. Furthermore, in all sections of the neocortex, we observed distinctive AT8ir granules, globules and threads in the molecular layer subjacent to the subpial TTR amyloid deposits. Thus, while we cannot be certain that all of the tau pathology in our case is not related to PART, these observations suggest the possibility of a link between the subpial amyloid and the neocortical tau pathology. Only rare neocortical neurons showing staining consistent with neurofibrillary tangles were seen, and overall, the tauopathy was much less severe than the neocortical tauopathy seen in patients with high AD neuropathology [24]. However, given the association of isocortical tauopathy/tangles with cognitive impairment in the elderly [31] and evidence supporting a role for pMAPT in the functional impairment of synapses [32], it is reasonable to hypothesize that the neocortical tauopathy observed in this case of meningovascular TTR amyloidosis contributed to the cognitive impairment. Further studies are needed to elucidate the prevalence and contribution of neocortical tauopathy to dementia in meningovascular amyloidosis, its potential relationship to PART and early AD neuropathologic changes, and a possible causal or contributory role for TTR amyloidosis in the neocortical tauopathy.

We evaluated for co-morbid dementia neuropathologies and found no evidence of Lewy bodies, TDP-43 proteinopathy or FUS proteinopathy. Ischemic lesions, which were reported in a kindred with an unspecified *TTR* mutation [33], were not prominent in our case. Our sections demonstrated only a solitary minute cortical microvascular ischemic lesion in the postcentral gyrus [31] and no evidence of ischemic white matter degeneration, suggesting that a significant burden of ischemic lesions is not necessary for dementia in meningovascular TTR amyloidosis. There was, however, evidence of vascular damage and chronic hemorrhage from the meningovascular amyloid leading to toxic superficial siderosis [23], which likely contributed to his cognitive impairment and ataxia.

## Conclusion

In summary, we have described the neuropathologic autopsy findings from a 72 year old male with variant Y69H *TTR* meningovascular amyloidosis. Our findings suggest that neocortical injury secondary to subpial TTR amyloid, injury to hippocampal efferent tracts secondary to subependymal TTR amyloid, and superficial siderosis may play important roles in the cognitive impairment and ataxia associated with variant *TTR* meningovascular amyloidosis. Our case, which demonstrated early PART, also showed neocortical tauopathy that was unusual for its subpial distribution. Further autopsy studies on patients with TTR meningovascular amyloidosis are necessary to elucidate the significance of tauopathy in the cognitive impairment and to further delineate the neuropathologic changes that underlie dementia. A better understanding of the pathogenic events leading to dementia in variant TTR meningovascular amyloidosis may lead to novel treatment strategies for this debilitating and fatal disease.

## Additional file

Additional file 1:
**Supplemental Material.** Supplemental Methods. **Table S1.** Vascular TTR amyloid in sampled systemic organs. **Table S2.** Comparison of TTR amyloid, pMAPT (AT8ir) and iron deposition across brain regions. **Figure S1.** Immunohistochemical stains did not label the amyloid protein. a. Amyloid-beta immunostains (clone 6F3D shown; clone 4G8 not shown) were negative in the meningovascular amyloid (Aβ; original magnification of 40×). b. A TTR immunostain is positive in the choroid plexus epithelium but does not convincingly label the vascular amyloid (TTR; original magnification of 40×). Staining of the intraventricular amyloid is equivocal. c. TEM image of leptomeninges (25,000×) demonstrating dense aggregates of amyloid fibrils. **Figure S2.** Molecular characterization of TTR amyloidosis. a. List of top 5 most abundant proteins detected by MS in amyloid samples taken from the leptomeninges of the prepontine cistern (Samples A1, A2) and cerebral hemispheres (Samples A4, A5). Note the abundance of transthyretin/TTR in the amyloid samples compared to control brain tissue samples of the basis pontis (Samples C3a, C3b) and subcortical white matter (SWM; Samples C6, C7). Interestingly, no transthyretin was detected in a core sample of the deep layers of the insular cortex (IC; Sample I) just subjacent to the subpial amyloid deposits demonstrated in Fig. 1d. Extracted ion chromatograms (EIC) of the normal and mutated transthyretin peptide T49-K70 for amyloid (A2, A4, A5) and control (C3, C6, C7) samples. Chromatographic peak areas under curves (AUC) suggest that approximately 85 %-90 % of the transthyretin protein in the amyloid regions exist in the mutated Y69H form corresponding to the mutant TTR allele. b. DNA sequencing results demonstrated the wild type TTR allele in 2 of 5 PCR clones from the cerebellar cortex and the mutant Y69H allele in 3 of 5 PCR clones. c. The APOE genotype of this patient, determined by restriction fragment length analysis, is E2/E3. Samples from this case (red font) were run alongside cases with known APOE genotypes (black font). **Figure S3.** Premortem 3T MRI images. a. FLAIR images demonstrating no evidence of ischemic subcortical leukoencephalopathy. The 3T images lack the resolution to examine for the white matter loss in the hippocampal outflow tracts (alveus, fimbria and fornix) seen by histology. b. GRE images demonstrating superficial siderosis in the cerebellar cortex and cerebrum (arrows). **Figure S4.** Limbic tauopathy was compatible with early primary age-related tauopathy (PART). AT8 immunoperoxidase stains demonstrated robust AT8ir pre-α cells of the entorhinal cortex (a, AT8; original magnification of 10×; b, AT8; original magnification of 100×) and few AT8ir neurons in the CA1 sector of the hippocampus (c, AT8; original magnification of 10×), compatible with Braak stage II (B1) PART. Prominent AT8ir threads were seen around the vestigial hippocampal sulcus (c). Entorhinal cortex tangles labeled with antibodies to 3R MAPT (d; original magnification of 100×) and 4R MAPT (e; original magnification of 100×), again compatible with PART. f. TEM image of entorhinal cortex neurofibrillary tangle (70,000×) from formalin-fixed tissue reprocessed for TEM revealing filamentous inclusions that are compatible with paired helical filaments. **Figure S5.** Additional neurodegeneration workup. a. Amyloid-beta immunostain (clone 4G8) demonstrated complete absence of neocortical plaques (4G8; original magnification of 200×). b. Very mild, focal cerebral amyloid-beta angiopathy was revealed by the 4G8 antibody (original magnification of 200×). c. α-synuclein immunostains revealed no cortical Lewy body neuropathology (α-synuclein; original magnification of 200×). d. TDP-43 immunostains revealed no abnormal threads or neuronal cytoplasmic or nuclear inclusions (TDP-43; original magnification of 200×). e. FUS immunostains revealed no abnormal neuronal cytoplasmic inclusions (FUS; original magnification of 200×). Positive control slides from AD, Lewy body disease, FTLD-U (TDP-43) and FTLD-U (FUS) were employed to demonstrate the efficacy of all of these antibodies (not shown). f. Only a single small remote cortical infarct was seen in a section of the precentral gyrus (H&E; original magnification of 10×). No other significant cerebrovascular disease pathology was present on standard dementia workup sections.
