# Supplementary material for: Neuropathologic analysis of Tyr69His TTR variant meningovascular amyloidosis with dementia
Source: Acta Neuropathol Commun. 2015 Jul 10;3:43. doi: 10.1186/s40478-015-0216-0 (PMC4496870; doi:10.1186/s40478-015-0216-0)
Supplement: Additional file 1: — Supplemental Material. Supplemental Methods. Table S1. Vascular TTR amyloid in sampled systemic organs. Table S2. Comparison of TTR amyloid, pMAPT (AT8ir) and iron deposition across brain regions. Figure S1. Immunohistochemical stains did not label the amyloid protein. a. Amyloid-beta immunostains (clone 6F3D shown; clone 4G8 not shown) were negative in the meningovascular amyloid (Aβ; original magnification of 40×). b. A TTR immunostain is positive in the choroid plexus epithelium but does not convincingly label the vascular amyloid (TTR; original magnification of 40×). Staining of the intraventricular amyloid is equivocal. c. TEM image of leptomeninges (25,000×) demonstrating dense aggregates of amyloid fibrils. Figure S2. Molecular characterization of TTR amyloidosis. a. List of top 5 most abundant proteins detected by MS in amyloid samples taken from the leptomeninges of the prepontine cistern (Samples A1, A2) and cerebral hemispheres (Samples A4, A5). Note the abundance of transthyretin/TTR in the amyloid samples compared to control brain tissue samples of the basis pontis (Samples C3a, C3b) and subcortical white matter (SWM; Samples C6, C7). Interestingly, no transthyretin was detected in a core sample of the deep layers of the insular cortex (IC; Sample I) just subjacent to the subpial amyloid deposits demonstrated in Fig. 1d. Extracted ion chromatograms (EIC) of the normal and mutated transthyretin peptide T49-K70 for amyloid (A2, A4, A5) and control (C3, C6, C7) samples. Chromatographic peak areas under curves (AUC) suggest that approximately 85 %-90 % of the transthyretin protein in the amyloid regions exist in the mutated Y69H form corresponding to the mutant TTR allele. b. DNA sequencing results demonstrated the wild type TTR allele in 2 of 5 PCR clones from the cerebellar cortex and the mutant Y69H allele in 3 of 5 PCR clones. c. The APOE genotype of this patient, determined by restriction fragment length analysis, is E2/E3. Samples from this case (red [file 40478_2015_216_MOESM1_ESM.docx]

**Supplemental Material**

**Supplemental Methods**

Histopathology

Brain tissues were fixed in 10% buffered formalin, processed for histology and embedded in paraffin. Tissue sections (6 μm thickness) were stained with hematoxylin and eosin. Additional sections underwent antigen retrieval with citrate buffer, were blocked with Power Block (Biogenex) and stained via immunoperoxidase reactions using diaminobenzidine chromogenic reactions (Biogenex). The following primary antibodies were used in the immunoperoxidase reactions: α-synuclein (Cell Signaling #2642, 1:1000); amyloid-beta (clone 6F/3D, Dako, M0872, 1:400; clone 4G8, BioLegend, SIG-39220, 1:500); Fused in sarcoma (FUS; Sigma, HPA008784, 1:3000); glial fibrillary acidic protein (GFAP; Dako, Z0334, 1:2000); myelin basic protein (Dako A0623, 1:400); phospho-MAPT (clone AT8; Thermo Scientific, MN1020, 1:2,000); 3-repeat isoform MAPT (3R MAPT, RD3; clone 8E6/C11, Millipore, 05-803, 1:250); 4-repeat isoform MAPT (4R MAPT, RD4; clone 1E1/A6, Millipore, 05-804, 1:250); TDP-43 (Proteintech 10782-2-AP, 1:10,000); TTR (Dako A0002, 1:4,000). All immunostains for protein inclusions were run alongside appropriate positive control cases. Immunoperoxidase stained slides were counterstained with hematoxylin. For Luxol fast blue-periodic acid Schiff (LFB-PAS) stains, slides were deparaffinized in xylene, hydrated and incubated in LFB solution (0.1 gm LFB in 100 ml 95% ethanol and 0.5 ml 10% glacial acetic acid). Slides were then rinsed and differentiated in 0.05% lithium carbonate followed by 70% ethanol. Slides were then placed in 0.5% periodic acid for 5 minutes, rinsed and developed in Schiff’s solution (American Master Tech) for 1 hour.

Additional stains for the assessment of neurodegenerative pathology were performed. For the modified Bielschowsky stain, slides were deparaffinized, hydrated and incubated in 20% silver nitrate for 30 minutes in a 37° oven. Following rinses in distilled water, slides were incubated in silver nitrate solution with ammonium hydroxide for 30 minutes at 37°, rinsed and incubated in silver nitrate solution with 1-2 drops of developer solution until the sections turned black with a golden-brown background. After several rinses the slides were incubated in 2% sodium thiosulfate, rinsed, dehydrated and coverslipped. For Gallyas silver stains, slides were deparaffinized in xylene, hydrated and incubated in 5% periodic acid. Following rinses in distilled water, slides were placed in alkaline iodide, washed in 0.5% acetic acid and incubated in developer solution containing anhydrous sodium carbonate, ammonium nitrate, silver nitrate, tungstosilicic acid and FORMORL until sections were pale brown-gray. The reaction was terminated in 0.5% acetic acid and slides were washed in acetic acid for 15 minutes. Slides were lightly counterstained with 0.5% Cresyl Violet, dehydrated and coverslipped.

Transmission Electron Microscopy (TEM)

Tissues were extracted from formalin-fixed, paraffin-embedded tissues. After soaking in xylene and re-hydrating in a graded ethanol series ending deionized water, tissues were fixed in ½ strength Karnovsky’s fixative in 0.1 M cacodylate buffer (pH 7.4) and post-fixed in 1% osmium tetroxide. Samples were then washed in deionized water and dehydrated in an acetone series, infiltrated with epoxy resin (modified Luft’s formulation), then embedded in fresh resin with catalyst and polymerized overnight. One micrometer sections for light microscopic review were stained with toluidine blue. Thin sections from areas of interest were cut at 70-90 nm on a Leica Ultracut S (Leica, Wetzlar, Germany), picked up on formvar coated grids (100 mesh Copper) and stained in 5% aqueous uranyl acetate followed by 1% aqueous lead citrate. Grids were observed using an Hitachi H7650 TEM at 80kV. Images were captured using an AMT XR-41 digital camera.

*APOE* Genotyping

Genomic DNA was extracted from tissue sections of the cerebellar hemisphere using the QiaAmp FFPE tissue kit (Qiagen #56404). *APOE* DNA was amplified by polymerase chain reaction using high fidelity DNA polymerase (Phusion), forward primer 5’-GCGGACATGGAGGACGTG-3’ and reverse primer 5’-GGCCTGGTACACTGCCAG-3’. PCR products were then digested with HhaI (New England Biolabs) and run on 10% TBS precast gels (Bio Rad, 456-5033) with ethidium bromide next to a 10 bp step ladder (Promega G4471). The *APOE* genotype was determined according to the method of Kamboh and colleagues [[15](#_ENREF_15)].

*TTR* Gene Sequencing

All 4 exons of the *TTR* gene were cloned from sections of the cerebellar cortex using Phusion high fidelity polymerase (Thermo Fisher, F-530S) and the following primer sets as previously described [[2](#_ENREF_2)]: 5’-CAGCAGGTTTGCAGTCAGAT-3’ and 5’-GGTACCCTTGCCCTAGTAAT-3’ for exon 1, 5’-CAATTTTGTTAACTTCTCACG-3’ and 5’-CAGATGATGTGAGCCTCTCTC-3’ for exon 2, 5’-CCTCCATGCGTAACTTAATCC-3’ and 5’-TAGGACATTTCTGTGGTACAC-3’ for exon 3, and 5’-TGGTGGAAATGGATCTGTCTG-3’ and 5’-TGGAAGGGACAATAAGGGAAT-3’ for exon 4. PCR products were gel purified, ligated into TOPO TA cloning vectors (Life Technologies, Cat # K4575J10), transformed into TOP10 competent cells (Life Technologies, Cat # K4575J10) and plated on agar plates containing ampicillin and Xgal dual selection. Five clones of each exon were sequenced.

Filter Aided Sample Preparation (FASP) and Mass Spectrometry (MS)

Tissue cores (3 mm diameter; 0.8 to 1.7 mg) punched from the formalin-fixed, paraffin embedded tissue blocks were analyzed by MS [[38](#_ENREF_38)]. The leptomeninges of the basal cisterns and cerebral convexities and brain parenchyma of the basis pontis, subcortical white matter and insular cortex were sampled in the tissue cores. The samples were deparaffinized in xylene and absolute ethanol, solubilized in 4% SDS and 100 mM Tris-HCL (pH 8.5), processed using a Precellys 24-Dual tissue homogenizer and incubated in a heating block at 99˚C for 60 minutes. Disulfide bonds were reduced via the addition of dithiothreitol to 100 mM at 55˚C for 20 min. The lysate was cleared by centrifugation at 10,000g for 10 minutes. Approximately 30% (30 uL) of each sample was then transferred to 30kDa centrifugal ultrafiltration filters and washed three times by centrifugation with 8 M Urea in 0.1 M Tris (pH 8.5) to remove the SDS as previously reported [[39](#_ENREF_39)]. Proteins were alkylated by adding iodoacetamide solution to the filter and incubated in the dark for 20 min, followed by centrifugation. This was followed with three washes of 8 M Urea, 0.1 M Tris-HCL pH 8.5, and three washes of 50 mM ammonium bicarbonate. Trypsin, at 1:100 protease to protein ratio, was added to the filter for overnight digestion at 37˚C. The peptides were eluted by centrifugation in 50 mM ammonium bicarbonate and 0.5 M sodium chloride, acidified with 50% formic acid in water, and concentrated in a C18 stage tip. The resulting peptides were loaded onto a Waters nanoAcquity Ultra Performance Liquid Chromatography (UPLC); the analytical column was pulled and packed in-house using 3μm C18 material. An LTQ Orbitrap Velos Mass Spectrometer in Data Dependent Acquisition mode fragmented the 15 most intense multiply charged precursor ions via collision induced dissociation. Data were searched against the Uniprot Human database using Sequest and Byonic, and results were filtered to 1% false discovery rate (FDR).

**Supplemental Table S1. Vascular TTR amyloid in sampled systemic organs.**

| **Organ** | **Vascular TTR Amyloidosis** |
| --- | --- |
| Heart (ventricular myocardium) | +++ |
| Liver (hepatic arteries) | +++ |
| Spleen | ++ |
| Kidney | ++ |
| Stomach | ++ |
| Lungs | + |
| Prostate | + |
| Table S1 Legend |  |
| Scale | Arterial Vessels Showing Mural Amyloid per Section |
| + | Focal |
| ++ | Multifocal |
| +++ | Universal |

**Supplemental Table S2. Comparison of TTR amyloid, pMAPT (AT8ir) and iron deposition across brain regions.**

| **Region** | **TTR Amyloid*** | **pMAPT (parenchymal/subpial)** | **Cerebral White Matter Pallor** | **Iron** |
| --- | --- | --- | --- | --- |
| Superior frontal gyrus | +++ | +++/++ | 0 | + |
| Middle frontal gyrus | +++ | +++/+++ | 0 | +++ |
| Inferior frontal gyrus | +++ | +/+ | 0 | + |
| Superior temporal gyrus | +++ | +/+ | 0 | ++ |
| Middle temporal gyrus | +++ | +/0 | 0 | ++ |
| Occipitotemporal gyrus | ++ | ++/++ | 0 | ++ |
| Insular cortex | +++ | ++/++ | 0 | +++ |
| Superior parietal lobule | +++ | +/++ | 0 | 0 |
| Inferior parietal lobule | +++ | +/+ | 0 | 0 |
| Parastriate cortex | + | +/0 | 0 | 0 |
| Striate cortex | +++ | +/++ | 0 | + |
| Precentral gyrus | +++ | ++/++ | 0 | + |
| Cingulate gyrus | ++ | ++/++ | 0 | 0 |
| Supracallosal gyrus | ++ | ++/+++ | N/A | 0 |
|  |  |  |  |  |
| CA1 | N/A | ++/N/A | N/A | 0 |
| Alveus/Fimbria/Fornix | +++ | + | +++ |  |
| Subiculum | +++ | +++/+++ | N/A | 0 |
| Fascia Dentata | N/A | 0/+++** | N/A | 0 |
| EC | +++ | +++/+++ | N/A | + |
| Amygdala | N/A | ++/N/A | N/A | 0 |
|  |  |  |  |  |
| Caudate nucleus | ++ | +/0 | N/A | + |
| Mammillary bodies | +++ | 0/+ | N/A | 0 |
| Nucleus basalis | N/A | +/N/A | N/A | 0 |
| Cerebellum | +++ | N/A/N/A | N/A | +++*** |
| * Extent of subpial or subependymal TTR amyloid deposits | | |  |  |
| ** AT8ir was very dense below the vestigial hippocampal sulcus | | |  |  |
| *** +++ iron was also seen in the cerebellar dentate nucleus | | |  |  |

Table S2 Legend

| Scale | TTR Amyloid | Gray Matter pTau (AT8; per hpf) | Subpial pTau (AT8) | Iron Aggregates |
| --- | --- | --- | --- | --- |
| 0 | None | None | None | None |
| + | Focal | 5 or less neurites and no neurons | Focal | less than 5 per hpf |
| ++ | Patchy | 6-10 neurites and/or 1-3 neurons | Patchy | 6-10 per hpf |
| +++ | Continuous | > 10 neurites and/or > 3 neurons | Continuous | greater than 10 per hpf |

**
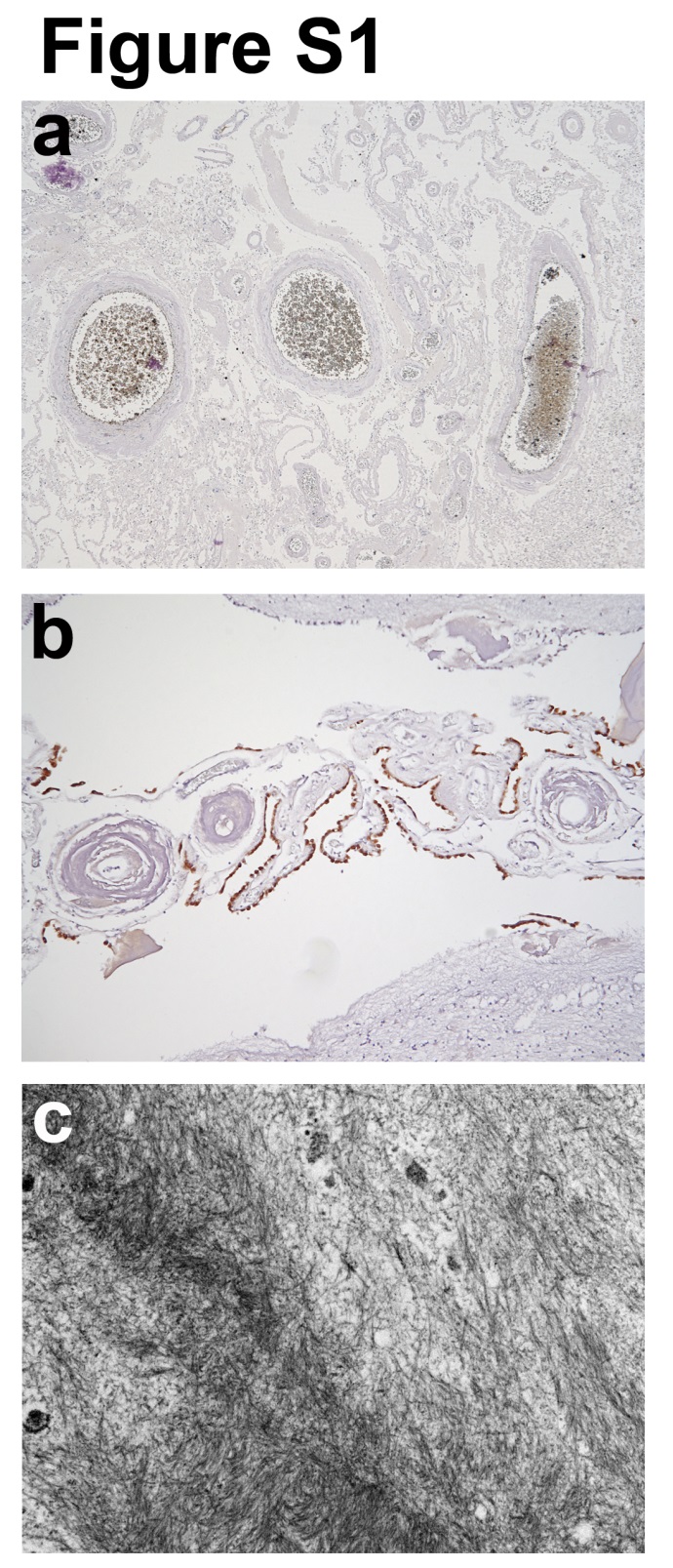
**

**Supplemental Figure S1. Immunohistochemical stains did not label the amyloid protein. a.** Amyloid-beta immunostains (clone 6F3D shown; clone 4G8 not shown) were negative in the meningovascular amyloid (Aβ; original magnification of 40x). **b.** A TTR immunostain is positive in the choroid plexus epithelium but does not convincingly label the vascular amyloid (TTR; original magnification of 40x). Staining of the intraventricular amyloid is equivocal. **c.** TEM image of leptomeninges (25,000X) demonstrating dense aggregates of amyloid fibrils.

**
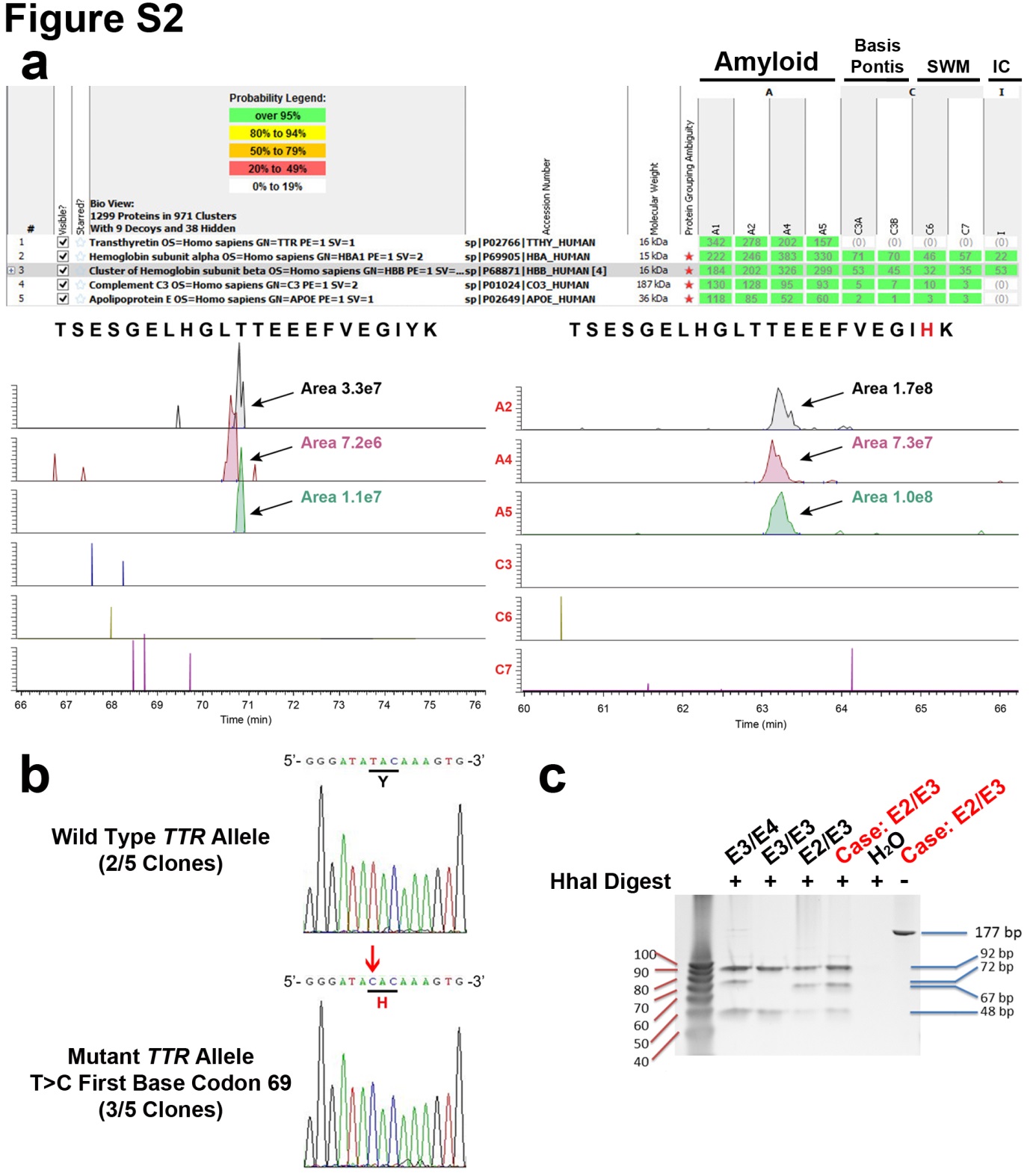
**

**Supplemental Figure S2. Molecular characterization of TTR amyloidosis. a.** List of top 5 most abundant proteins detected by MS in amyloid samples taken from the leptomeninges of the prepontine cistern (Samples A1, A2) and cerebral hemispheres (Samples A4, A5). Note the abundance of transthyretin/TTR in the amyloid samples compared to control brain tissue samples of the basis pontis (Samples C3a, C3b) and subcortical white matter (SWM; Samples C6, C7). Interestingly, no transthyretin was detected in a core sample of the deep layers of the insular cortex (IC; Sample I) just subjacent to the subpial amyloid deposits demonstrated in Fig. 1d. Extracted ion chromatograms (EIC) of the normal and mutated transthyretin peptide T49-K70 for amyloid (A2, A4, A5) and control (C3, C6, C7) samples. Chromatographic peak areas under curves (AUC) suggest that approximately 85% -90% of the transthyretin protein in the amyloid regions exist in the mutated Y69H form corresponding to the mutant *TTR* allele. **b.** DNA sequencing results demonstrated the wild type TTR allele in 2 of 5 PCR clones from the cerebellar cortex and the mutant Y69H allele in 3 of 5 PCR clones. **c.** The *APOE* genotype of this patient, determined by restriction fragment length analysis, is E2/E3. Samples from this case (red font) were run alongside cases with known *APOE* genotypes (black font).


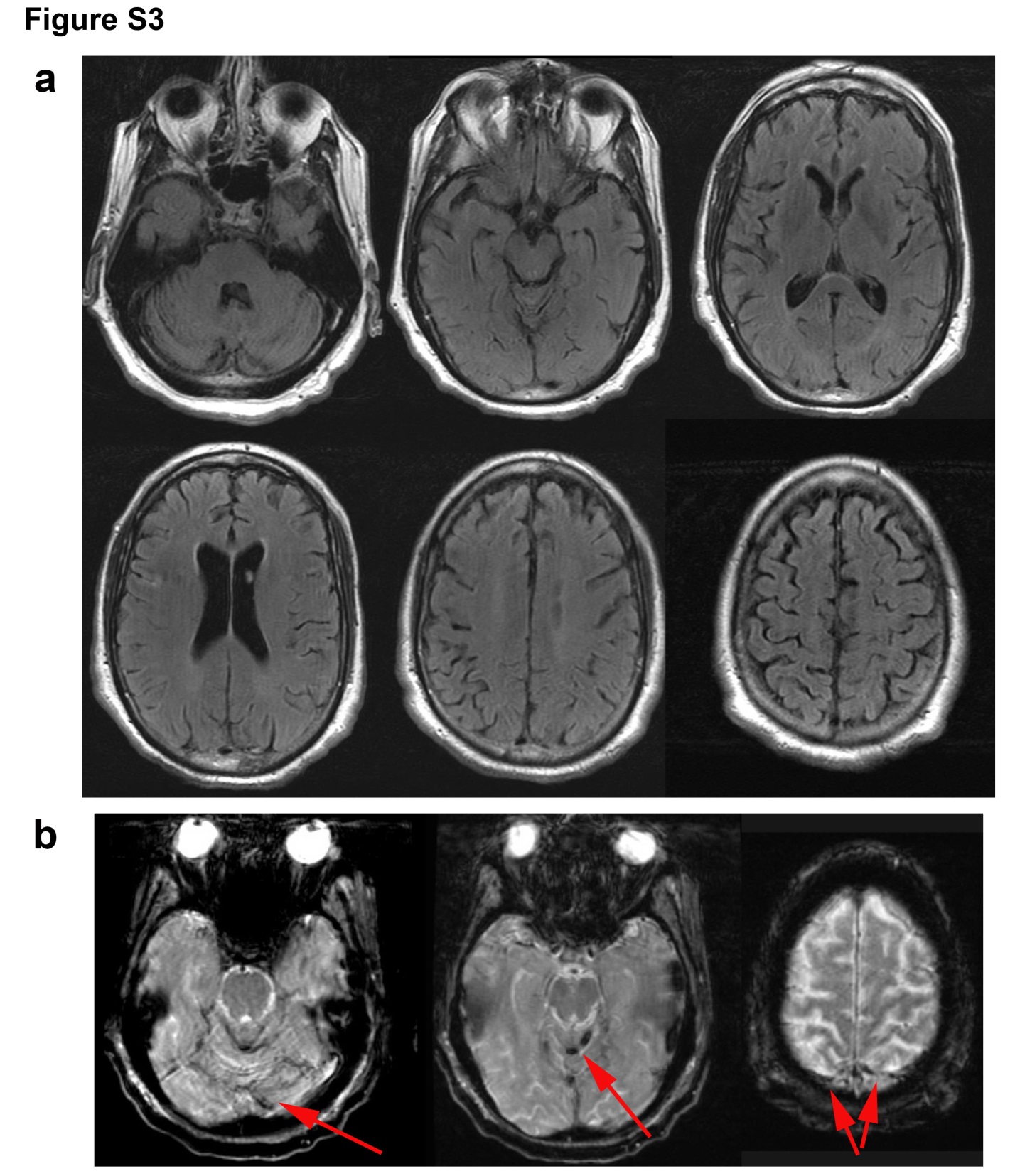


**Supplemental Figure S3. Premortem 3T MRI images. a.** FLAIR images demonstrating no evidence of ischemic subcortical leukoencephalopathy. The 3T images lack the resolution to examine for the white matter loss in the hippocampal outflow tracts (alveus, fimbria and fornix) seen by histology. **b.** GRE images demonstrating superficial siderosis in the cerebellar cortex and cerebrum (arrows).

**
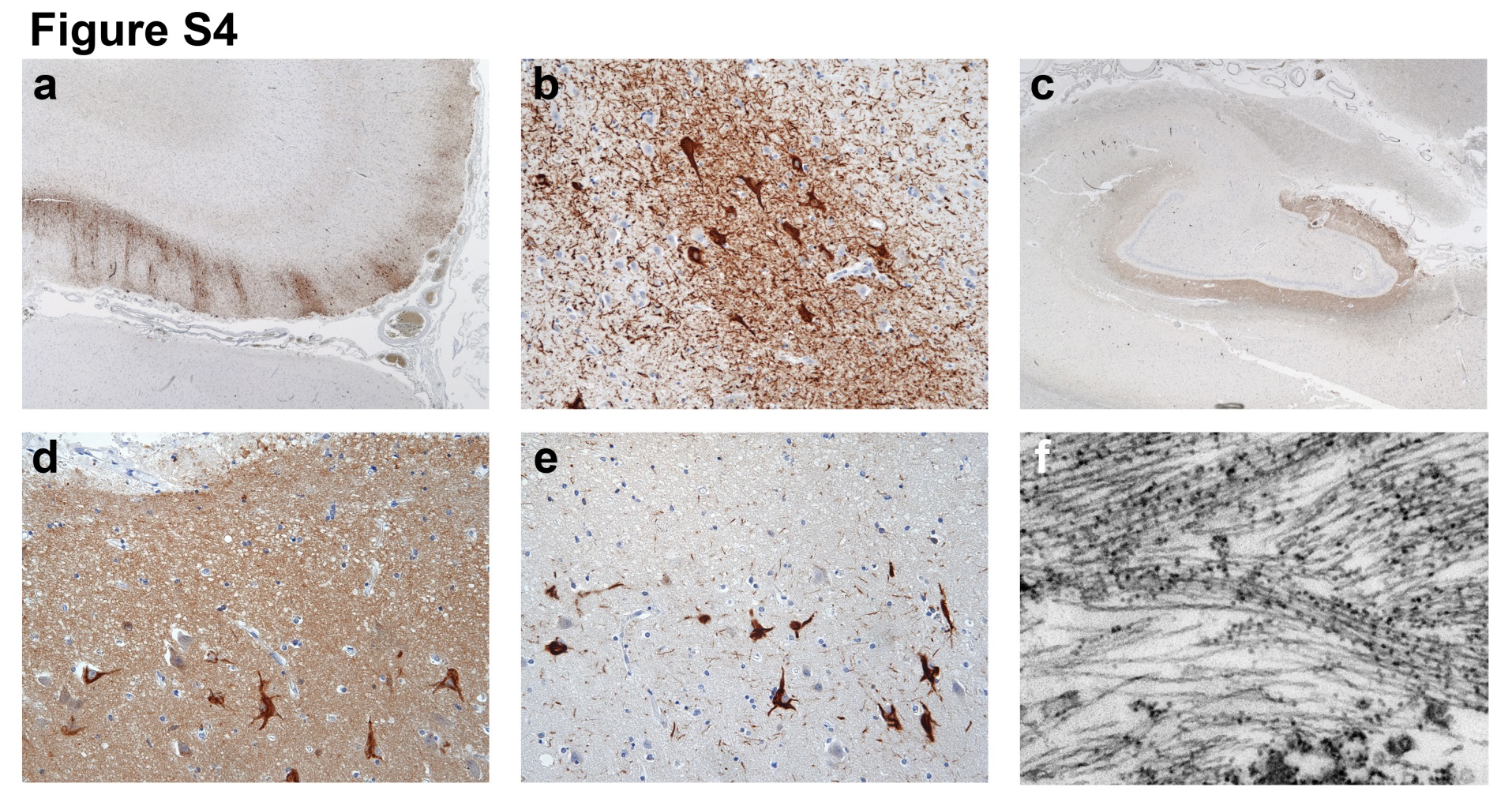
**

**Supplemental Figure S4. Limbic tauopathy was compatible with early primary age-related tauopathy (PART).** AT8 immunoperoxidase stains demonstrated robust AT8ir pre-α cells of the entorhinal cortex (**a,** AT8; original magnification of 10x**; b,** AT8; original magnification of 100x) and few AT8ir neurons in the CA1 sector of the hippocampus (**c,** AT8; original magnification of 10x), compatible with Braak stage II (B1) PART. Prominent AT8ir threads were seen around the vestigial hippocampal sulcus (c). Entorhinal cortex tangles labeled with antibodies to 3R MAPT (**d**; original magnification of 100x) and 4R MAPT (**e**; original magnification of 100x), again compatible with PART. **f.** TEM image of entorhinal cortex neurofibrillary tangle (70,000X) from formalin-fixed tissue reprocessed for TEM revealing filamentous inclusions that are compatible with paired helical filaments.

**
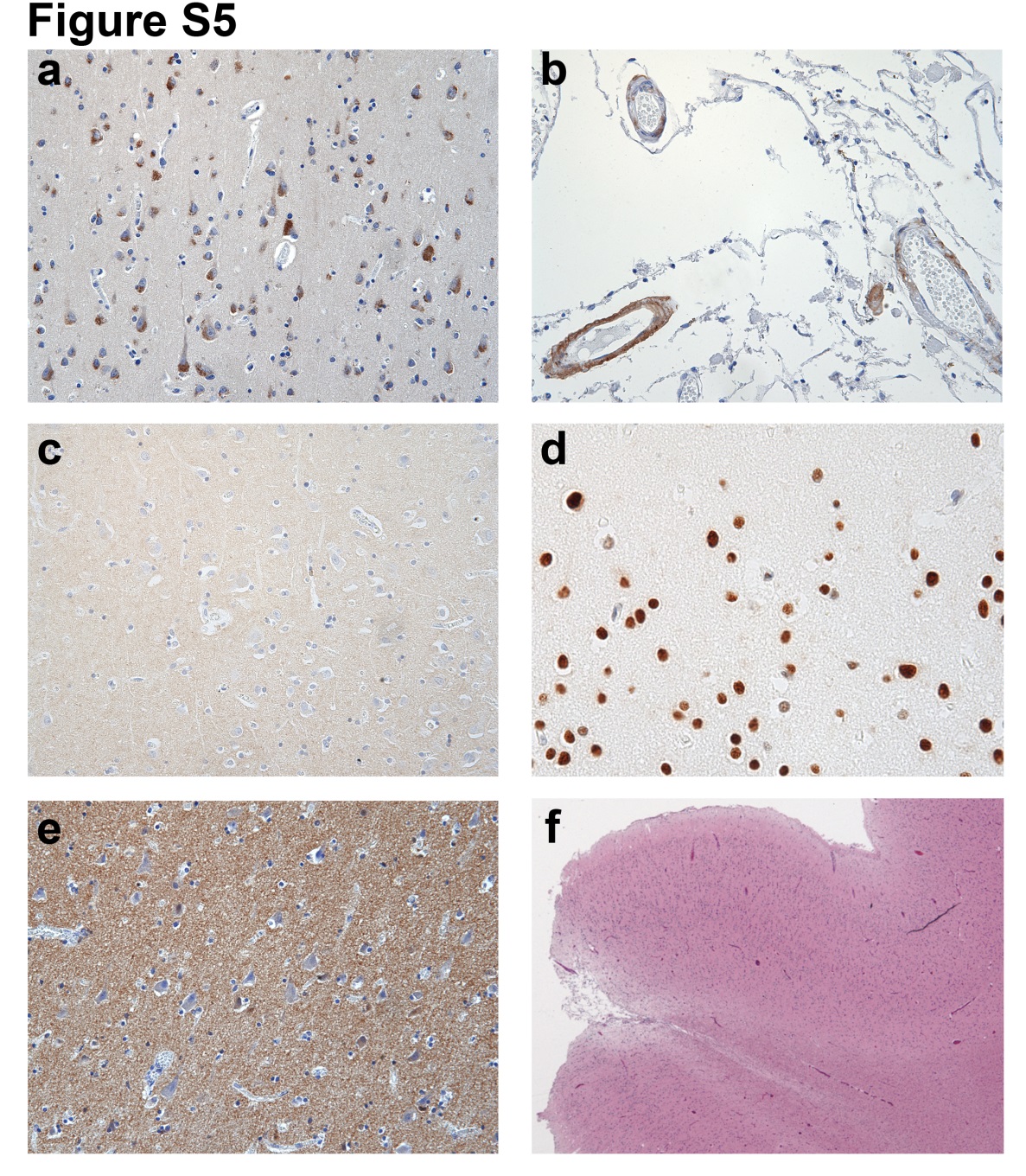
**

**Supplemental Figure S5. Additional neurodegeneration workup. a.** Amyloid-beta immunostain (clone 4G8) demonstrated complete absence of neocortical plaques (4G8; original magnification of 200x). **b.** Very mild, focal cerebral amyloid-beta angiopathy was revealed by the 4G8 antibody (original magnification of 200x). **c.** α-synuclein immunostains revealed no cortical Lewy body neuropathology (α-synuclein; original magnification of 200x). **d.** TDP-43 immunostains revealed no abnormal threads or neuronal cytoplasmic or nuclear inclusions (TDP-43; original magnification of 200x). **e.** FUS immunostains revealed no abnormal neuronal cytoplasmic inclusions (FUS; original magnification of 200x). Positive control slides from AD, Lewy body disease, FTLD-U (TDP-43) and FTLD-U (FUS) were employed to demonstrate the efficacy of all of these antibodies (not shown). **f**. Only a single small remote cortical infarct was seen in a section of the precentral gyrus (H&E; original magnification of 10x). No other significant cerebrovascular disease pathology was present on standard dementia workup sections.
